# Evidence-based severity assessment of the forced swim test in the rat

**DOI:** 10.1371/journal.pone.0292816

**Published:** 2023-10-12

**Authors:** Laura Becker, Anne S. Mallien, Natascha Pfeiffer, Christiane Brandwein, Steven R. Talbot, André Bleich, Rupert Palme, Heidrun Potschka, Peter Gass

**Affiliations:** 1 Department of Psychiatry and Psychotherapy, RG Animal Models in Psychiatry, Central Institute of Mental Health, Medical Faculty Mannheim, Heidelberg University, Mannheim, Germany; 2 Hannover Medical School, Institute for Laboratory Animal Science, Hannover, Germany; 3 Department of Biomedical Sciences, University of Veterinary Medicine, Vienna, Austria; 4 Institute of Pharmacology, Toxicology, and Pharmacy, Ludwig-Maximilians-University, Munich, Germany; University of Modena and Reggio Emilia, ITALY

## Abstract

The forced swim test (FST) is a traditional assay, which has been used for more than 40 years to assess antidepressant effects of novel drug candidates. In recent years, a debate about the test has focused on the assumption that the FST is highly aversive and burdening for the animals because of the earlier anthropomorphic interpretation and designation as a "behavioral despair test". The Directive 2010/63/EU and the German Animal Welfare law require a prospective severity classification of the planned experimental procedures. Still, an objective examination of the animals’ burden in this test has not been performed yet. To fill this gap, we conducted an evidence-based severity assessment of the forced swim test in rats according to a ’standard protocol’ with a water temperature of 25°C. We examined parameters representing the physiological and the affective state, and natural as well as locomotion-associated behaviors in three separate experiments to reflect as many dimensions as possible of the animal’s condition in the test. Hypothermia was the only effect observed in all animals exposed to the FST when using this standard protocol. Additional adverse effects on body weight, food consumption, and fecal corticosterone metabolite concentrations occurred in response to administration of the antidepressant imipramine, which is frequently used as positive control when testing for antidepressant effects of new substances. We conclude that this version of the FST itself is less severe for the animals than assumed, and we suggest a severity classification of ’moderate’ because of the acute and short-lasting effects of hypothermia. To refine the FST according to the 3Rs, we encourage confirming the predictive validity in warmer water temperatures to allow the rats to maintain physiological body temperature.

## Introduction

The forced swim test (FST), also known as the Porsolt test, has been used for over 40 years to detect novel antidepressant agents [1]. It is a widely known and discussed behavioral test for different reasons: the model’s validity, its terminologies and use, and as an example for harm-benefit analyses of ethical problems in laboratory animal science.

A short time after the development of the FST, a debate on the terminology of the immobility readout as ‘behavioral despair’ emerged [2]. Further criticism points to the increased use of the FST as a model for depression, although the test’s validity, especially construct validity, has been highly questioned [3–6].

Instead of interpreting it as despair, these authors argue that the immobility in the forced swim test is a passive coping mechanism of rodents confronted with an inescapable stressor and a learned response to conserve energy [2, 4–6]. The discussion on immobility readouts reflects that the regulation of FST behavior is still not understood [6, 7].

Nevertheless, as summarized in several reviews, the predictive validity of the FST has been proven in various studies reporting an impact of licensed antidepressants on the immobility response in mice and rats [8–11]. However, such reviews and the underlying original publications should be considered with caution in light of the negative publication bias [12, 13]. Nonetheless, existing evidence for a predictive validity and the apparent lack of validated alternative testing methods, the scientific field should not hastily give up on the FST.

Another debate focuses on the harm caused by the test. Following the anthropomorphic interpretation of floating as behavioral despair, the FST has ever since ensured media attention. Animal rights organizations criticize the FST for inducing ‘fear, anxiety, terror, and depression in small animals’ [14, 15]. Only until a few years ago, the global number of publications using the forced swim test was increasing, but to the best of the authors’ knowledge, no study has yet focused on the assessment of severity to the animals in the FST [6], apart from measuring corticosterone and other stress hormones related to the swim exposure [16].

In Europe, the Directive 2010/63/EU regulates animal experimentation and requires a prospective severity classification for all planned experimental procedures in the categories ‘mild’, ‘moderate’, ‘severe’, and ‘non-recovery’. An exemplary catalog is provided in appendix 8 but only classifies ‘forced swim tests […] with exhaustion as the end-point’ as a severe experiment and lacks an estimation for tests limited in time (Directive 2010/63/EU). The laboratory animal science association, the British Association for Psychopharmacology, the Physiological Society, and Understanding Animal Research have published a fact sheet on the forced swim test proposing a severity classification of ‘moderate’ [17]. However, an evidence-based assessment of severity is lacking.

To close this gap, we set up an experimental design to objectively measure the impacts of the FST on different kinds of behavior in rats, following a suggestion of the UK National Centre for the Replacement, Refinement, and Reduction of Animals in Research (NC3Rs) [18]. We decided to analyse the severity of the procedure in rats. Of course, the severity can species-specific: rats are heavier than mice and might have more difficulties to float effortlessly on the water surface, but are also less prone to hypothermia and have a higher preference to swimming than mice. We used parameters that explore the physiological and affective state, natural and homecage behaviors, and locomotion to investigate many dimensions of the animal’s experience. To reflect the severity in as many laboratories as possible, we decided to conduct a ‘standard’ version of the FST as described by Slattery and Cryan [19].

## Material & methods

### Experimental plan

**Fig 1:**
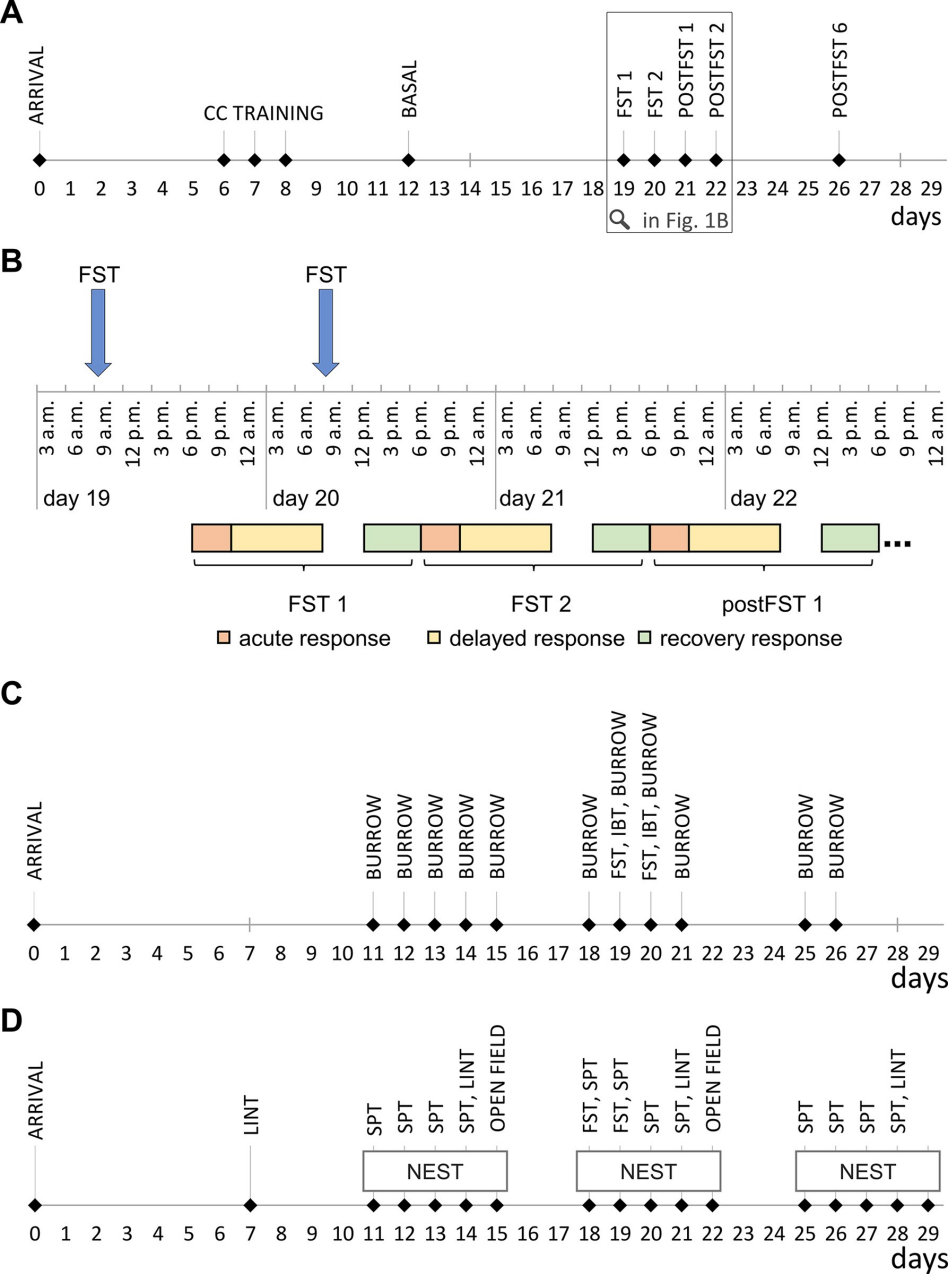
(A-D): Experimental design. All three experiments were conducted in male and female animals. (A) Timeline of the FCM experiment. We used n = 8/group in the first male cohort and n = 10/group in the second male cohort. The first female cohort consisted of n = 10/group and the second n = 8/group. CC = Cage change training. (B) Sampling scheme in the FCM experiment. The detailed chronological sequence of the collection intervals is shown exemplarily for the days 19 to 22. The ‘acute response’ interval went from 10 h to 13.5 h after the FST, leading to collection times from 7 pm to 10:30 pm. The ‘delayed response’ interval was from 10:30 pm to 9 am the next day, i.e., 13.5–24 h after the FST. ‘Recovery response’ samples were timed 28–34 h after an FST session, therefore between 1 pm and 7 pm (C) Timeline of the Burrowing experiment. The group size was n = 12 for both sexes. Because of poor burrowing behavior in male animals, only data for female rats are available. BURROW = burrowing, IBT = inner body temperature. (D) Experimental timeline of the homecage parameters and the open field. We used n = 12/group in both sexes. LINT = latency until interaction with nest material test, SPT = saccharin preference test, NEST = nest score.

For assessing the severity of the Forced Swim Test (FST) in the rat, we conducted three experiments, each examining separate parameters indicating welfare impairments to reduce the risk of interference between parameter measurements. Parameters were studied at three different time points to allow for within-subject comparisons additional to between-subject comparisons: at a baseline level, in the week of the FST, and one week following the FST, to assess potential recovery effects. The timelines are shown in Fig 1A–1D. In experiment 1, we investigated the impact of the FST on fecal corticosterone metabolite levels (FCM) (Fig 1B). Effects of the FST on burrowing behavior and inner body temperature (IBT) were tested in experiment 2 (Fig 1C). Non-invasive parameters (nest score, latency until interaction with nest material test (LINT), saccharin preference test) in the homecage and locomotion tested in the open field (OF) were analyzed in experiment 3 (Fig 1D). All experiments were performed according to the regulations for animal experimentation in the European Union (European Communities Council Directive 2010/63/EU) and in the German Animal Welfare Act and were approved by the German animal welfare authorities (Regierungspräsidium Karlsruhe, 35-9185-81-G-15-21). Reporting of this project follows the ARRIVE 2.0 guidelines [20].

### Animals

We investigated the impact of the forced swim test on female and male Wistar-Han rats. We used male rats purchased from Charles River (Sulzfeld, Germany) in the FCM experiments and in the burrowing experiment. In all other experiments, rats bred by the Janvier Labs (Le Genest-Saint-Isle, France) were used. At arrival, the animals were eight weeks old and were handled for five minutes five times a week. We randomized the assignment to the treatment groups. We comparatively analyzed four treatment groups: 1) rats that swam in the FST and were intraperitoneally (i.p.) treated with imipramine (FST-Imi), 2) rats that swam in the FST and were injected with saline (FST-Sal), 3) rats that were exposed to the FST without any injection (FST) and 4) a mere handling control group (Ctrl). 5) An additional group of animals was used to consider the circadian variations of corticosterone levels (DV) in experiment 1. The rats were housed pairwise in type IV cages (Tecniplast, Hohenpeißenberg, Germany), equipped with bedding (Abedd Espen MIDI, ABEDD, Vienna, Austria), sizzle nest, gnawing sticks (both Zoonlab, Castrop-Rauxel, Germany) and unbleached cellulose tissue paper at a 12 h light-dark cycle (7 am– 7 pm, unless stated otherwise). Cages were changed weekly unless otherwise indicated. Food (LASQCdiet Rod16, Altromin Spezialfutter GmbH & Co. KG, Lage, Germany) and tap water were provided *ad libitum*, and their intakes were measured weekly or daily, depending on the experiment. The body weight of the rats was measured five times a week in all cohorts between 8 and 9 o’clock in the morning. We performed additional measurements in female rats (17 weeks old, n = 5) to assess the time until the animals regain their physiological body temperature after the 15 min swim procedure. Experiments with male and female animals were performed at separate time points to avoid pheromonal effects on animal behavior. In general, the experimenters remained blinded to the treatment. However, it was not possible in some instances as the same experimenters performed the FST and the well-being tests.

### Forced swim test

The beakers for the FST consisted of transparent acrylic glass cylinders (height: 50 cm, inner diameter: 19 cm). They were filled to the height of 30 cm with 25°C warm water. The beakers were filled with fresh water for each animal. The testing took place 2–3.5 h after the onset of light. The experimental room was dimly lit with 15 lux. Up to four animals were tested simultaneously after acclimatizing to the room for approximately 20 min. They could not see each other during swimming as dark grey dividers were placed between the beakers. The forced swim test was conducted over two consecutive days. On day 1, the swimming duration was 15 min, and on day 2 they swam for 5 min, as published by Slattery and Cryan [19]. After swimming, the rats were towel-dried and placed back in their homecages. The cages were warmed with red light for approximately 30 min after the FST.

### Drugs

The Imipramine was injected intraperitoneally (i.p.) 1 h and 23.5 h after the first swim at a dose of 10 mg/kg imipramine (Sigma-Aldrich Chemie GmbH, Taufkirchen, Deutschland). Imipramine was dissolved in a volume of 10 mg/ml in sterile saline the day before usage. The vehicle control group (FST-Sal) was injected with an equal volume of sterile saline at the same time points as FST-Imi rats.

### Handling control

Rats assigned to the control group were acclimatized to an experimental room, different from the room where the FST was conducted, for approximately 20 min. The dry rats were then towel-dried for one minute to control for handling influences. Afterward, they were transferred back to their homecages. We omitted red-light exposure for these control animals, as this may have overheated the animals because they did not experience the hypothermia due to the water exposure.

#### Experiment 1: FCM measurement

Rat feces were collected on multiple days at three pre-defined time points, referred to as ‘acute response’, ‘delayed response’, and ‘recovery response’. According to Lepschy et al. [21], the fecal corticosterone metabolites peak after 10–12 h following an ACTH stimulation. The exact times of the collection intervals are based on the FST times: ‘Acute response’ samples were obtained 10–13.5 h after the FST and were followed by collecting the ‘delayed response’ samples 13.5–24 h. Finally, the ‘recovery response’ was collected 28–34 h after the FST (Fig 1A and 1B). The three different FCM intervals were collected at a basal time point and on the first FST day, the second FST day, and the day after. Additionally, 2 and 6 days after the FST protocol, another ‘acute response’ sample was collected to rule out a persistent elevation of corticosterone. Two cohorts for each sex were used to examine FCM concentrations (total group size in both sexes was n = 18). Each cohort was divided into two batches, housed in separate rooms with time-shifted light cycles (batch 1: lights on at 7 am, batch 2: lights on at 8:30 am) to maintain the same time intervals and therefore take the diurnal variation of the corticosterone metabolism into account during experimentation.

For the exact timing of the intervals, the rats were transferred to a different set of ‘collection cages’ (equally sized and enriched as the housing cages but with reduced bedding). Two collection cages were assigned to each pair of animals, in addition to the regular homecage, to keep their environment familiar. All fecal pellets were collected before each new use of the respective cages. To avoid stress induction by frequent cage changes, the animals were already trained in the habituation phase to frequent handling and cage changes at the sample times (Fig 1B).

The collected feces were kept at -20°C until further processing. The feces were then dried for 5 h in an incubator at 65°C and subsequently homogenized. The samples were further processed as described in Mallien et al. [22]. Briefly, 50 mg of homogenized, powdered feces were mixed with 1 ml of 80% of methanol and shaken for 30 min. Finally, the methanolic extract was centrifuged for 10 min before taking the supernatant for further analysis, as described Lepschy and colleagues [21].

#### Experiment 2: Burrowing and inner body temperature measurement

The burrowing assay was conducted as previously described [22–25]. Shortly, the rats were trained to burrow in a separate experimental room for five days. On day one, the animals were only acclimatized to a type IV cage laid out with unbleached cellulose paper tissues and an empty burrowing tube (32 cm long x 10 cm in diameter, open end 6 cm elevated from the floor) for 30 min. On days 2–5, the burrowing tube was exchanged for another burrowing tube filled with gravel (2000 g) after acclimatization. After 60 min, the rats were transferred back to their homecages, and the remaining gravel in the tubes was measured. On day 5, the latency to start burrowing was additionally analyzed. Rats that did not begin burrowing in the first 10 min were assigned a latency of 600 s for the statistical analysis. Between trials, the gravel was washed with 0.1% acetic acid. The tubes and cages were cleaned with 70% ethanol. Seventeen out of 48 rats burrowed less than 500 g on day 5 and were excluded from the analysis as described elsewhere [26].

After training, the burrowing behavior of the rats was tested one day before the FST, on both FST days, the day after the FST, and 5 and 6 days after the FST for severity assessment (Fig 1C). Directly after the FST or the respective handling procedure, the inner body temperature of the animals was measured rectally (Rodent thermometer BIO-TK8851, Bioseb Lab Instruments, France).

#### Experiment 3: Homecage-based parameters and open field

In the third experiment, non-invasive homecage parameters, like the nest score, the latency until interaction with the nest material test (LINT), the saccharin preference test, and locomotion, were examined (Fig 1D).

Nest-building behavior was assessed five times a week for three consecutive weeks. The nests were scored 2 h after the end of the dark phase and according to the method previously published by Schwabe et al. [27] and Mallien and colleagues [22]. In summary, ‘0’ was assigned in case the nest material was almost untouched, ‘1’ was given when the nest material was touched and distributed over 50% of the floor area without a visible nest area; a score of ‘3’ was rated for nests that showed a marked nest area which could either be flat with an indentation or present an appreciable height; the highest score of ‘4’ was assigned for perfect nests: both a noticeable height as well as a prominent indentation needed to be visible [27]. The fresh nesting material was placed in the same position in each cage change.

The LINT was measured during the weekly cage change. In the male cohort, the rats were placed in the new cage, already equipped with sizzle nest material (formed into two balls, each weighing 14 g), paper, and the gnawing stick. Then, the time was measured until the animals interacted with the nest material for more than 3 s, as described in Möller et al. [26]. We counted carrying, digging, and gnawing the material as interactions. Animals that did not display an interaction after 600 s were assigned 600 s as latency as a cut-off point. For the female animals, we changed the protocol changed slightly because we observed that in the previous examined males, many rats did not show interest in the nest material immediately after cage change but were instead occupied with playing with the cage mate and/or playing with the fresh bedding or the gnawing stick. Therefore, the female animals were transferred to a new cage only filled with bedding for one hour to acclimatize. Then, 28±1 g of sizzle material, formed into two balls, was added to the cage, and the latency was measured as previously described. After the LINT, paper, and gnawing sticks were placed in the cages.

Locomotion of the animals in an open field (OF) was analyzed for 30 min twice at the interval of one week. Before the conduction of the OF, the rats were transferred to an experimental room and acclimatized for 30 min. Four animals were tested in 4 adjacent boxes (50 cm in length × 50 cm in width × 50 cm in height), dimly lit with 25 lux simultaneously. After each trial, the apparatus was cleaned with 70% ethanol to prevent odor cues. We analyzed the total distance moved (TDM), center time (CT), velocity, and movement in 5 min time bins after automatic tracking (Ethovision XT 15, Noldus Information Technology, Wageningen, Netherlands) [22, 24, 28].

The saccharin preference test SPT was repeatedly conducted for three consecutive weeks according to the protocol by Klein et al. [29]. On days 1 and 3, the animals were presented with two water bottles to compare the regular consumption, check for a side preference, and prevent habituation to the sweet taste. On days 2 and 4, one bottle was filled with 0.1% saccharin solution (Sigma-Aldrich Chemie GmbH, Taufkirchen, Deutschland), while the other bottle contained plain water. The position of the saccharin bottle was switched with the water bottle on both days to control for side preference. 24 h values were measured for both cohorts. An additional 2 h measurement was evaluated in the female cohort because we did not observe any treatment effects in male animals tested before the female animals. A dripping control, i.e., an additional cage equipped with two bottles, was used to correct the fluid loss due to cage handling. This cage was removed from the rack and opened like all other animal cages. Animals consuming the same or less fluid in 2 h than lost by the dripping control (mean of all 2 h measurements) were excluded from this analysis. This affected 2 from 24 cages in the 2 h measurement.

#### Statistical data analysis

Statistical analysis was performed using R [30] using the packages lme4 [31] and lmerTest [32]. Data were tested against the hypothesis of normal distribution using the Shapiro-Wilk test. Data from male and female animals and cohorts were analyzed separately whenever sex interactions or cohort interactions were present. Body weight, water and food consumption, amount of burrowed gravel, inner body temperature, and open field outcomes were analyzed using linear models (resp. parameter ~ treatment + time + treatment:time). To assess the time until the animals regained their physiological body temperature after swimming we used the following linear model (temperature ~ time). For the analysis of FCM, body weight change, and saccharin preference, linear mixed-effects models were used because of single missing data points and/or improved residual distribution. We chose animal ID, cage (in cage-based parameters) and if appropriate cohort as random effects. Non-parametrical testing was performed using the Kruskal-Wallis Test. We compared two time points with the Wilcoxon-Test to analyze the nest scores. Multiple group comparisons were corrected with the Tukey post hoc test. The experimental unit was the single animal, except for SPT, FCM, water consumption, food consumption, and nest score, where the cage was used as the experimental unit. We used G*power *a priori* analysis to calculate the sample size based on estimated effect sizes for one-way ANOVA [33]. For data visualization, the R package ggplot2 was utilized [34].

## Results

Our multimodal approach for severity assessment combined parameters from physiological, affective, locomotion-associated, and intrinsic natural behaviors.

### Impact of the FST on physiological parameters

#### Body weight change

**Fig 2:**
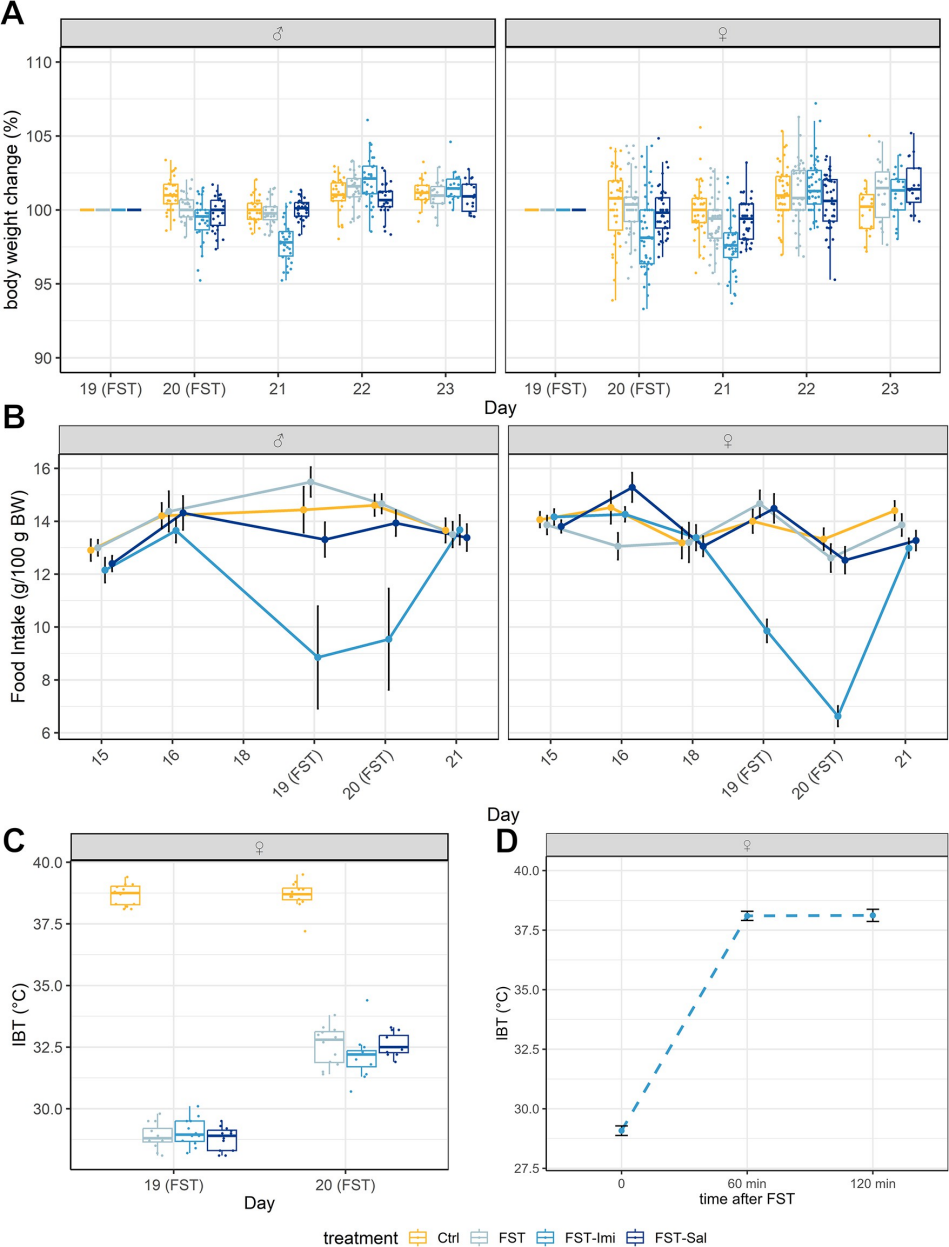
(A-D) Physiological parameters. (A) Body weight change. The percentage change in body weight from the previous day during the week of the FST is shown as a boxplot, separated by sex. Animals of the FST-Imipramine group show a mild but significant loss of body weight after the FST and Imipramine injections. Data from all cohorts were considered. Sample size of male animals n = 30 for all treatment groups. Sample size of female animals was n = 42 for each treatment group. (B) Food intake. The mean and standard error of the daily food consumption per 100 g body weight is displayed separately for male and female rats. Rats treated with Imipramine showed a significantly reduced food consumption on the days of the injections. The intake of the FST and FST-Sal groups did not change. Sample size of male animals: n = 5 cages per treatment group. Sample size of female animals: n = 8–20 cages per treatment group. (C) Inner body temperature. Boxplots showing the internal body temperature measured directly after the forced swim test/the handling procedure on both FST days. The forced swim test led to a significant drop in temperature. Only data for female animals are available. Sample size: n = 12 per treatment group. (D) Recovery to physiological body temperature. Already after 60 min the female rats could regain their normal body temperature. Sample size: n = 5.

Analysis of the body weight change (BWC) did not reveal general treatment effects (male animals: F(3, 530) = 0, p = 1, female animals: F(3, 721.02) = 0, p = 1) but time:treatment interactions (male animals: F(12, 530) = 13.45, p<0.001; female animals: F(12, 721.02) = 5.77, p<0.001) in both sexes (Fig 2A–2D). A cohort effect was present in both sexes, but because of similar effects, the datasets of the cohorts were combined. In male animals, the control group gained weight after the pretest of the FST (day 20) compared to all other treatment groups, and the FST-Imipramine group showed a lower body weight gain compared to the FST group (P_adj_: Ctrl vs. FST-Imi p<0.001, Ctrl vs. FST p = 0.003, Ctrl vs. FST-Sal p<0.001, FST vs. FST-Imi p = 0.005). After the second FST (day 21), the FST-Imi group showed a loss of body weight (mean ± SE, 97.9 ± 0.19), unlike all other treatment groups (P_adj_: Ctrl vs. FST-Imi p<0.001, FST vs. FST-Imi p<0.001, FST-Sal vs. FST-Imi p<0.001). On day 22, the FST-Imi compensated the small body weight loss again and showed a higher BWC compared to the control group and the FST-Sal group as well as the FST-group compared to the FST-Sal animals (P_adj_: Ctrl vs. FST-Imi p<0.001, FST vs. FST-Sal p = 0.03, FST-Imi vs. FST-Sal p<0.001). In female animals, the FST-Imi group lost weight on day 20 (mean ± SE, 98.4 ± 0.26) and on day 21 (mean ± SE, 97.6 ± 0.26) (P_adj_, day 20: Ctrl vs. FST-Imi p<0.001, FST vs. FST-Imi p<0.001, FST-Sal vs. FST-Imi p<0.001, day 21: Ctrl vs. FST-Imi p<0.001, FST vs. FST-Imi p<0.001, FST-Sal vs. FST-Imi p<0.001). On day 22, the rats in the FST-Imi group compensated the weight loss and showed a positive BWC that was higher than of the FST-Sal animals (P_adj:_ p = 0.04). The FST-Sal animals gained more weight on day 23 than the control group (P_adj:_ p = 0.03).

We did not observe significant treatment effects or time:treatment interactions of the forced swim test on the absolute body weight in the week of the FST in male and female animals (data shown in S1 File). Although not statistically significant, a kink in the growth curve could be seen in all cohorts and sexes in the FST-Imi group.

#### Inner body temperature

Measurement of the inner body temperature showed that all female animals swimming in the FST suffered a pronounced drop in temperature on both days (FST 1: F(3, 44) = 1122, p<0.001; FST 2: F(3, 44) = 238.4, p<0.001; P_adj_: Ctrl vs. FST p<0.001, Ctrl vs. FST-Imi p<0.001, Ctrl vs. FST-Sal p<0.001; Fig 2C). The estimated mean±SE for the FST group was 28.9±0.15°C, for the FST-Imi group 29.1±0.15°C and the FST-Sal animals 28.8±0.15°C after the 15 min FST pretest. After the 5 min swim on the next day, the drop of the inner body temperature was lower but still pronounced: FST group 32.6±0.2°C, for the FST-Imi group 32.1±0.2°C and the FST-Sal animals 32.6±0.2°C. The experiment performed to assess the recovery after the 15 min swim, revealed that the female rats regained their physiological body temperature already after 60 min (Fig 2D). The estimated mean±SE directly after the swimming was 29.1±0.22°C, and 38.1±0.22°C after 60 min as well as after 120 min (F(2, 12) = 571, p<0.001, P_adj_: 0 min vs. 60 min p<0.001, 0 min vs. 120 min p<0.001). No data for male animals are available.

#### Food consumption

Analysis of the daily food intake revealed a time:treatment interaction. Male and female animal in the FST-Imi group ate less on both FST days compared to all other treatment groups (male animals: F(12, 79) = 03.11, p = 0.001; female animals: F(15, 387) = 13.1, p<0.001), indicating a pharmacological effect of Imipramine (Fig 2B). Two measurements (day 15 and day 16) of female animals were excluded from the analysis using the 3σ-rule; one measurement was higher and the other lower compared to the mean of the other animals. The intercept for male animals was 12.91 g (SE 0.79 g), and for female animals, 14.15 g (SE 0.56 g). When we measured the food consumption weekly (male FCM1 and HC+OF cohort), we could not detect any effects, probably because the imipramine effect only lasted for two days (data shown in S1 File).

#### Water intake

Measuring the water intake once a week did not show any effects in male or female animals (data shown in S1 File). As for food consumption, the lack of effect may be related to the long interval. Analysis of the water consumption in 24 h on the ‘control days’ of the saccharin preference test only showed a non-significant reduction of water intake in the FST-Imipramine animals (male: F(3, 39) = 2.2, p = 0.1; female: F(3, 40) = 1.5, p = 0.23).

#### Fecal corticosterone metabolites (FCMs)

**Fig 3:**
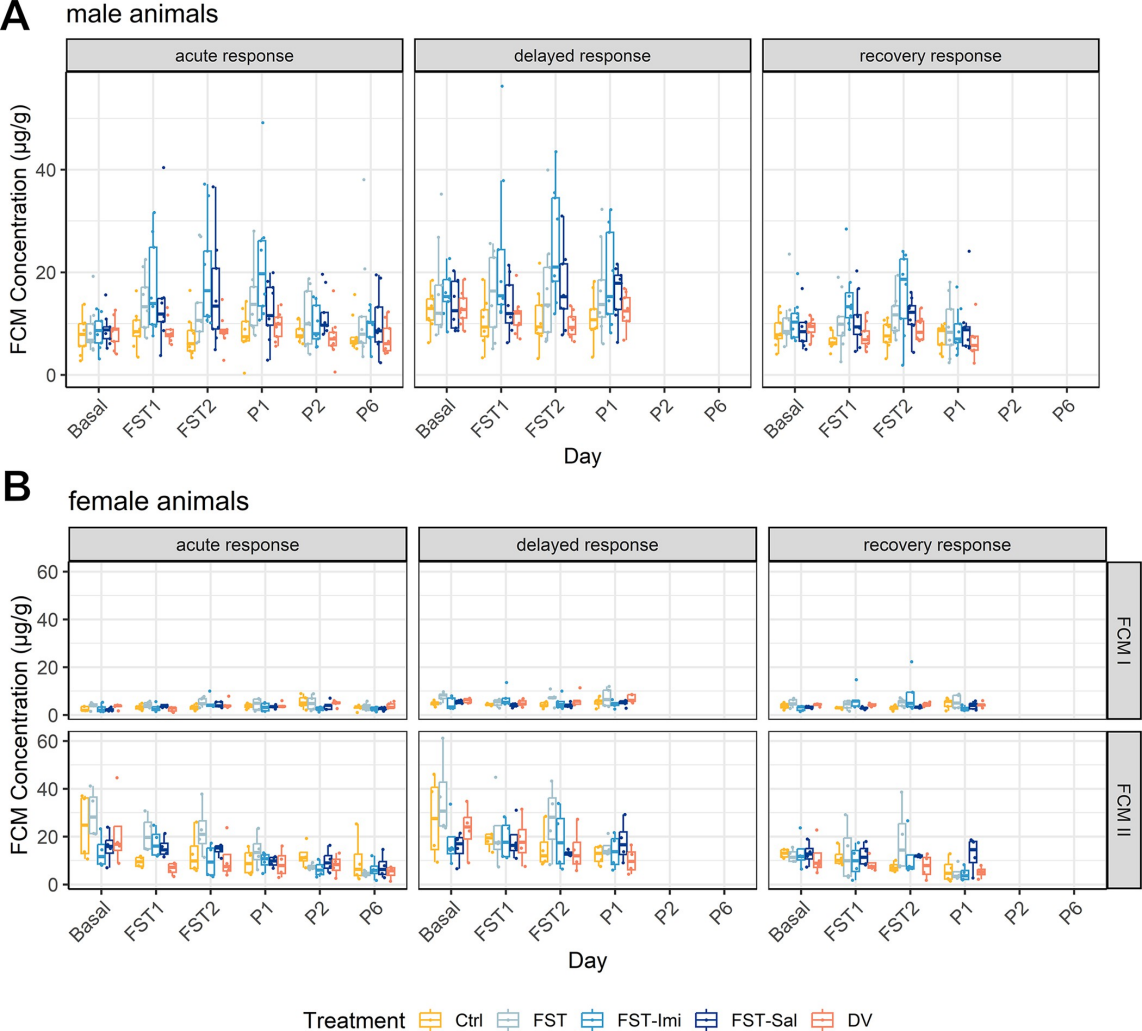
(A-B) Fecal corticosterone metabolite concentrations are shown as boxplots. (A) FCM response in male animals. The panels show the FCM concentrations of all tested days separated by the collection intervals ‘acute response’, ‘delayed response’, and ‘recovery response’. Animals of the FST-Imi group displayed higher concentrations at the time points FST1, FST2, and P1 in the ‘acute response’ interval and at FST1 and FST2 during the ‘recovery response’. No other treatment group showed significantly elevated stress hormone metabolite levels. The sample size was n = 17-18/group. (B) FCM response in female animals. The FCM concentration in female animals showed substantial cohort differences. Therefore, the panels are separated by cohort and collection interval. In the first cohort (FCM I), we could detect elevated FCM levels after the second FST in the FST-Imi group during the ‘recovery response’. In cohort two (FCM II), animals displayed much higher FCM concentrations, especially during the baseline measurement. In the ‘acute response’ interval, the Control-group showed higher FCM values than the FST-Imi group at P2, two days after the FST. No significant time:treatment interactions could be detected in this cohort. The sample size in the first cohort was n = 10/group, and in the second, n = 8/group.

In male animals, we observed time:treatment interactions for the ‘acute response’ (F(20, 200) = 1.7, p = 0.03) and ‘recovery response’ (F(12, 119.1) = 2, p = 0.02) but no general treatment effects (Fig 3A). The acute response of the FST-Imi group was characterized by elevated FCM concentrations after the 15 min FST pretest (P_adj_: Ctrl vs. Imi p = 0.06, DV vs. FST-Imi p = 0.04). One day later, after the 5 min FST test, the FST-Imi group continued to show elevated FCM concentrations compared to the handling and diurnal variation control (P_adj_: Ctrl vs. FST-Imi p<0.001, DV vs. FST-Imi p = 0.003). On P1, the imipramine-treated animals still had significantly higher FCM concentrations than almost all other groups (P_adj_: FST-Imi vs. Ctrl. p<0.001, FST-Imi vs. DV p<0.001, FST-Imi vs. FST p = 0.07, FST-Imi vs. FST-Sal p = 0.02). In the ‘recovery response’ interval, the FST-Imipramine group also presented elevated FCM concentrations after both FST days compared to handling and DV control (P_adj_: FST-Imi vs. Ctrl p = 0.008, FST-Imi vs. DV p = 0.03 on FST1; FST-Imi vs. Ctrl p = 0.003, FST-Imi vs. DV p = 0.01 on FST2).

The FCM analysis in female animals demonstrated a strong cohort effect, so both cohorts were analyzed separately (Fig 3B). Generally, the measured FCM concentrations in cohort FCM2 were exceptionally high. In the first cohort (FCM 1), we did not observe general treatment effects, but a time effect in the ‘acute response’ interval (F(5, 100) = 3.9, p = 0.003) and a time:treatment interaction in the first interval (F(20, 100) = 1.7, p = 0.046) as well as in the ‘recovery response’ interval (F(12, 60) = 3.1, p = 0.002). The handling group showed higher FCM concentrations than the FST-Imi group two days after the FST (P_adj_: FST-Imi vs. Ctrl p = 0.03) in the ‘acute response’ interval. During the ‘recovery response’ on the day of the second FST (FST2), the Imipramine group displayed increased levels of FCM in contrast to the handling and FST-Saline animals (FST-Imi vs. Ctrl p = 0.005, FST-Imi vs. FST-Sal p = 0.009). Analysis in the second FCM cohort (FCM 2) did not reveal a treatment effect or time:treatment interactions during all sample intervals but a time effect in the ‘acute response’ interval (F(5, 75) = 3.02, p = 0.01), showing that FCM concentrations were higher in basal samples compared to all other sampling time points (P_adj_: Basal vs. FST1 p = 0.01, Basal vs. FST2 p = 0.02, Basal vs. P1 p<0.0001, Basal vs. P2 p<0.0001, Basal vs. P6 p<0.0001), indicating stress during the baseline measurement. Additionally, the levels of FCM samples were lower on P6 compared to FST1 and FST2 (P_adj_: FST1 vs. P6 p = 0.01, FST2 vs. P6 p = 0.008).

### Impact of the FST on the affective state

#### SPT

**Fig 4:**
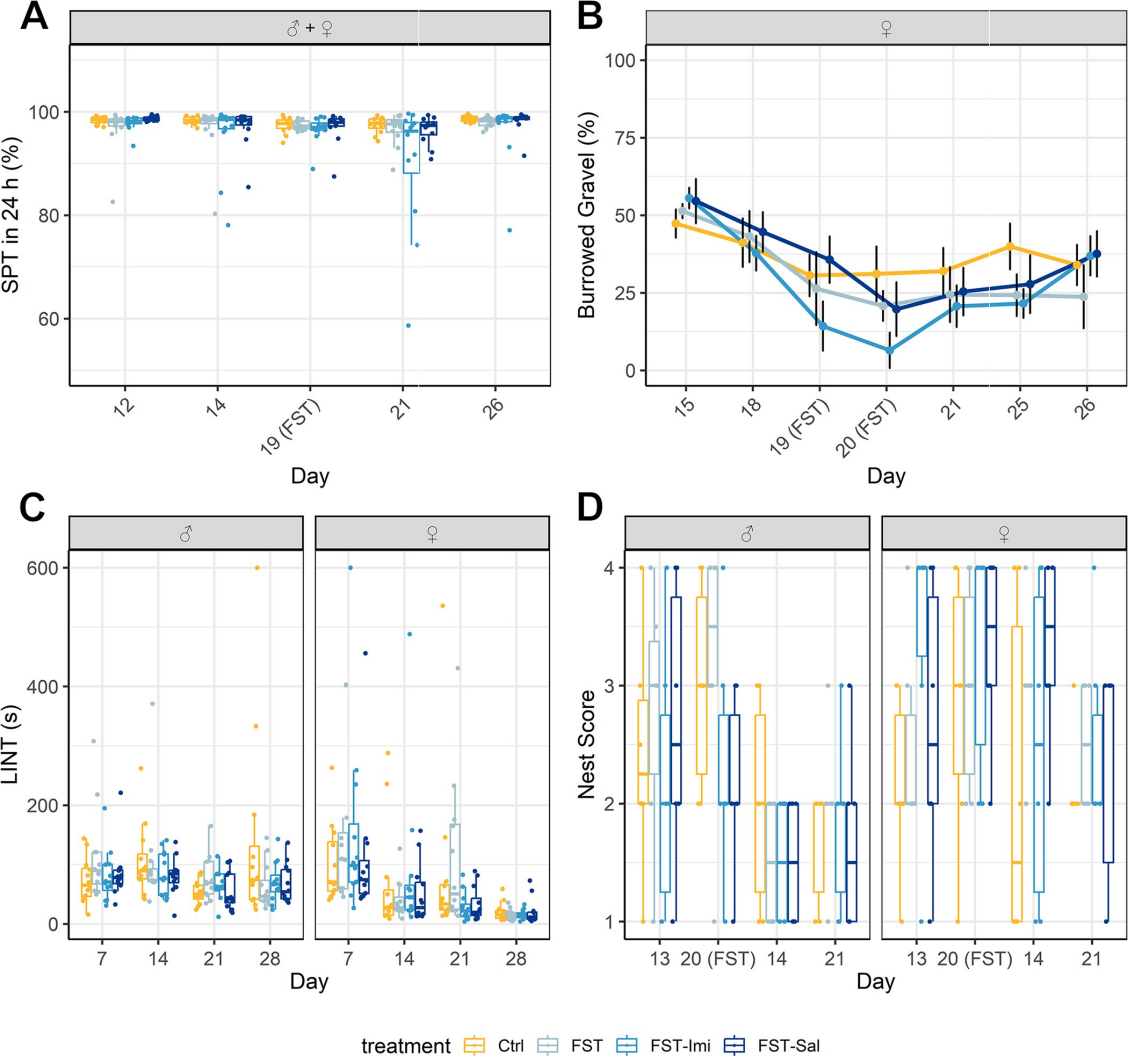
(A-D) Species-specific behaviors. (A) Saccharine preference test results over 24 h are shown as a boxplot. No significant time:treatment interaction was observed in the 24 h saccharin preference test. N = 6 cages per treatment group. (B) Burrowed gravel in the burrowing assay. The mean amount of burrowed gravel (± SEM) is depicted from the last training day (D15). We did not find significant treatment effects or time:treatment interactions. Sample sizes were: Control n = 8, FST n = 6, FST-Imi n = 9, FST-Sal n = 8. (C) Latency until interaction with nest material test displayed as a boxplot. No effects of the FST were observed. The sample size for all groups was n = 12. (D) Nest Score. Baseline nest scores on days 13 and 14 are shown together with the scores on the mornings after the two FST days (day 20 and day 21) as boxplots. The sample size for all groups was n = 6 (cage-based parameter) for each sex.

The 24 h test of the saccharine preference did not show a sex effect. Therefore the datasets of females and males were combined (Fig 4A). All treatment groups demonstrated a high preference for saccharin. The intercept was 98.34% (SE 1.22%). Neither a treatment effect (F(3, 204.9) = 0.48, p = 0.69) or a time:treatment interaction (F(12, 176) = 1.5, p = 0.13) were found but a higher variation in the preference of the FST-Imi animals one day after the FST was visible. To increase the sensitivity of the saccharin preference test, we added a measurement in the female cohort two hours after the FST. In a shorter interval (two hours after FST) no group showed a decreased saccharin preference (treatment effect: F(3, 98) = 0.12, p = 0.95, time:treatment interaction: F(12, 78.6) = 1.2, p = 0.29; figure shown in S1 File). Intake values that lay below the mean of the drip control were excluded from the 2 h analysis, as we did not assume a representative preference for these (2 cages out of 24).

### Impact of the FST on natural behaviors

#### Burrowing

No treatment effect (F(3, 189) = 0.29, p = 0.84) or time:treatment interaction (F(18, 189) = 0.74, p = 0.76) could be detected in the analysis of the relative amount burrowed by female rats (Fig 4B). The latency until the start of the burrowing behavior was at no point significantly different for the respective treatment groups (data in S1 File).

The male rats used in our experiment did not burrow well enough to establish a stable baseline. Twenty-one animals, resp. 44% of the cohort did not burrow more than 500 g on training day 5. Of the remaining 27 rats, some animals showed high intra-individual variances, i.e., they burrowed one day and not the following day. This phenomenon was also observed in a study by Whittaker and colleagues [35] and Riedesel et al. [25]. We have also tried to let the rats burrow in pairs, consisting of one animal that did not perform well and one that burrowed reasonable amounts, as recommended in one study [36], but this did not improve the performance. In addition, a prolongation of the training for additional 4 days did not lead to improved burrowing performance, so we decided to stop the experiment.

#### Nest score

Analysis of nest-building behavior in male and female rats revealed that the forced swim test did not lead to altered nest scores. The Wilcoxon test did not show significant differences in the mornings after the FST (data in S1 File). Since the nest scores depend on the day of the week [27], the nests of the respective weekdays of the baseline week were compared with the FST week. Nest scores of male and female animals were analyzed separately. The nest scores of the two days after the FST compared to the baseline (day 13 vs. day 20; day 14 vs. day 21) are shown in Fig 4D.

#### LINT

In female and male animals, the latency until interaction with the nest material test did not differ among the treatment groups during the entire study (data in S1 File, Fig 4C).

### Impact of the FST on locomotion

#### Open field

**Fig 5:**
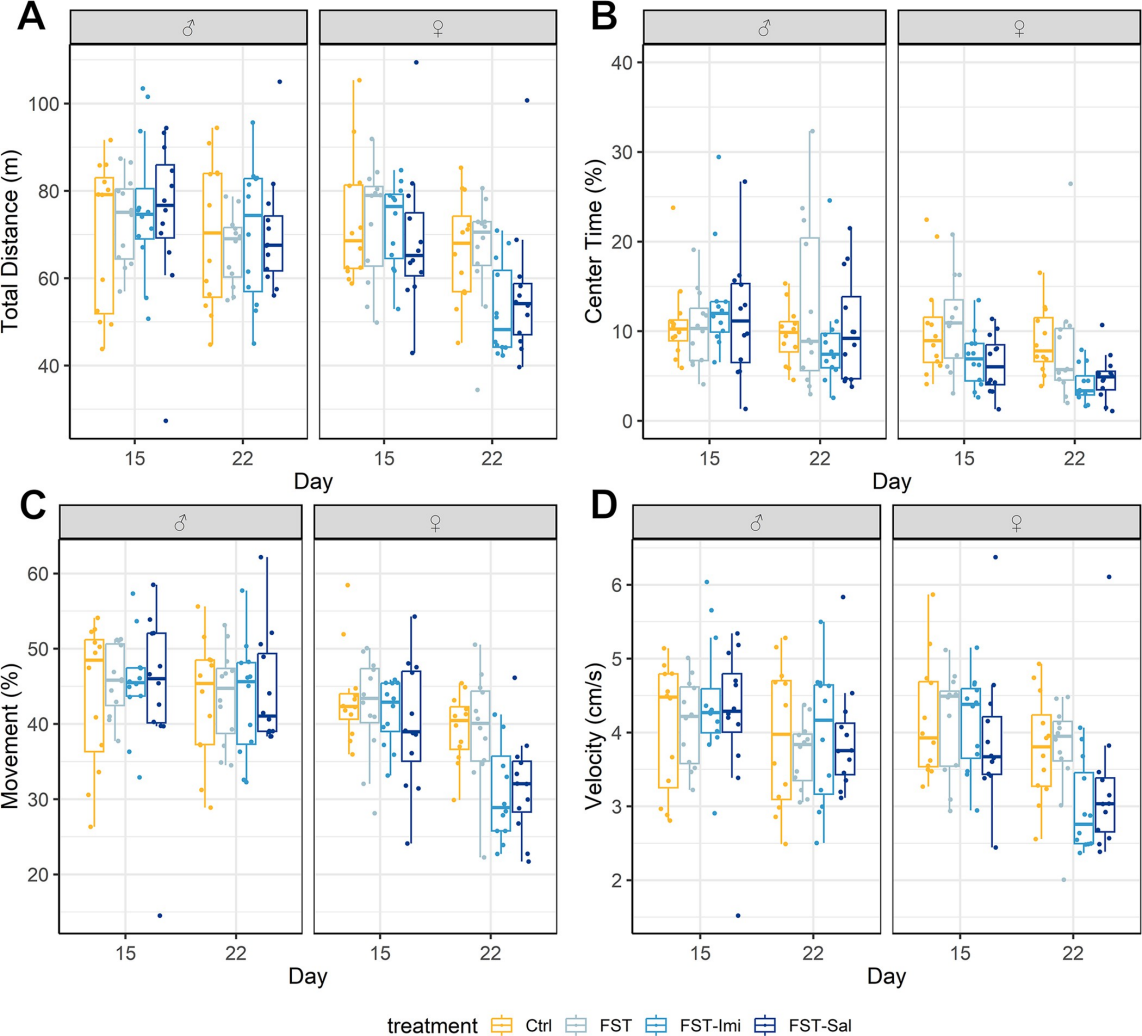
(A-D) Open field. All parameters are shown as boxplots and separately for female and male animals. Results from the baseline week (day 15) and after the FST (day 22) are displayed next to each other. We did not see effects in any of the open field parameters. The group size was n = 12 per sex. (A) Total distance moved over the time course of 30 min. (B) Center time. (C) Relative movement. (D) Velocity.

None of the parameters analyzed in the open field demonstrated time:treatment interactions in both sexes (Fig 5A–5D). The total distance moved during the 30 min was not influenced by the FST, as we did not observe significant time:treatment interactions in male (F(3, 88) = 0.26, p = 0.86) and female (F(3, 88) = 1.3, p = 0.27) animals (Fig 5A). Also, no treatment effects were observed in both sexes (male: F(3, 88) = 0.37, p = 0.78, female: F(3, 88) = 0.27, p = 0.85). The time the animals spent in the center of the open field did not show time:treatment interactions as well (male: F(3, 88) = 1.3, p = 0.27, female: F(3, 88) = 0.19, p = 0.9, Fig 5B). The female animals showed a general treatment effect (F(3, 88) = 3.7, p = 0.01), which occurred because of an unstable baseline. We did not find time:treatment interactions in the velocity in both cohorts (male: F(3, 88) = 0.2, p = 0.9, female: F(3, 88) = 1.32, p = 0.27, Fig 5D). The relative proportion in the movement has also not changed as a result of the FST (time:treatment interaction—male: F(3, 88) = 0.1, p = 0.96, female: F(3, 88) = 1.67, p = 0.18, Fig 5C). We did not observe time effects in any parameter, indicating that no habituation effects resulted from repeating the open field after one week.

A brief summary of the outcomes of all analyzed parameters can be found in Table 1.

**Table 1:** Summary of results.

Parameter	Sex	Outcome*
Body weight	♂	n.s.
	♀	n.s.
Body weight change	♂	FST-Imi: Lower body weight gain after the FST pretest, short-lasting loss of body weight after FST (5 min)
	♀	FST-Imi: short-lasting weight loss after both FST days for two days
Water consumption (1x weekly)	♂	n.s.
	♀	n.s.
Water consumption (1 h after FST)	♂	n.s.
	♀	n.s.
Food consumption (1x weekly)	♂	n.s.
	♀	No data
Food consumption (1x daily)	♂	FST-Imi: Less food consumption on both FST (+ Imipramine inj.) days
	♀	FST-Imi: Less food consumption on both FST (+ Imipramine inj.) days
FCM	♂	FST-Imi: higher FCM concentrations in the ‘acute response’ interval, and in the ‘recovery response’ interval after the FST
	♀	Strong cohort effect. Cohort 1: Ctrl group showed higher FCM concentration than the FST-Imi group two days after the FST in the ‘acute interval’. The FST-Imi group had higher FCM concentrations than the Ctrl and FST-Sal groups on the day of FST2 in the ‘recovery response’. Cohort 2: time effect–baseline values higher
Burrowing	♂	No data
	♀	n.s.
Inner body temperature	♂	No data
	♀	FST, FST-Sal, FST-Imi: Pronounced loss of temperature on both FST days
Nest Score	♂	n.s.
	♀	n.s.
LINT	♂	n.s.
	♀	n.s.
Open field	♂	n.s.
	♀	n.s.
Saccharin preference	♂+♀	n.s.

*More details can be found in the results section under the respective parameter.

## Discussion

This study investigated the burden of the forced swim test, a traditional assay for assessment of antidepressant effects. The impact on the general condition and the affective state was assessed in male and female rats. Therefore, we chose parameters examining physiological aspects and affective state as well as species-specific, and locomotion behavior. Following evidence-based assessment of the severity of the FST, we planned to determine suitable refinement measures according to the 3R principle [37]. Interestingly, the only effect observed in all FST groups was hypothermia after the FST. Animals swimming in the FST and additionally treated with Imipramine (FST-Imi) showed additional effects on body weight, FCM, and food intake.

Cooling due to the 25°C tempered water seems to be the most relevant effect of the forced swim test on animal welfare due to the small body volume. We measured the temperature in female rats, but several studies performed in male rats did confirm hypothermia after the FST [16, 38–40]. Water temperatures of 15°C and 19°C decreased homecage or exploratory behavior directly after swimming, whereas 25°C cold water or warmer water temperatures did not have this effect [38, 41]. Rewarming in a warm water bath diminished the effect of reduced activity after a swim procedure [41]. In two publications, the application of imipramine at a dosage of 15 and 30 mg/kg led to an additive temperature loss [16, 39], a finding we could not confirm using a dosage of 10 mg/kg. All these findings highlight the importance of drying and warming the animals after the forced swim test to quickly regain body temperature homeostasis. In our opinion, the drying and rewarming should be an inherent part of any forced swim procedure. We could show that the animals have recovered from hypothermia already 60 min after the 15 min forced swimming and after being dried with towels and rewarmed for approximately 30 min with red light. In the previously mentioned study, animals regained a normal core body temperature after 90 min after the 15 min forced swim in 24 ± 1°C cold water [16]. The physical consequences of hypothermia should not be overlooked: Several studies showed detrimental effects of hypothermia on spatial working memory and damage in the hippocampus [42, 43]. A possible refinement of the forced swim test might therefore be using warmer water to prevent hypothermia, which is only rarely done so far [44, 45]. However, several studies report higher floating times when the water temperature is near thermoneutrality [16, 38, 46]. Therefore, the pharmacological validity of the FST must be confirmed at higher temperatures.

Regarding the body weight, we observed a short growth stagnation in male rats after the first day of the FST compared to the handling control, but no significant weight loss due to the FST on its own. On the contrary, female and male rats additionally treated with imipramine were found to lose body weight (<5%) on the two days after imipramine application but recovered quickly. We assume that the weight loss resulted from reduced food intake, which occurred in the FST-Imipramine groups only. This has already been demonstrated in one study [47] during chronic imipramine administration and displays a pharmacological effect.

In humans several side effects of imipramine such as e.g. dizziness, constipation, loss of appetite, xerostomia (dry mouth), nausea and weight gain frequently occur. Whether imipramine primarily influences central regulation of appetite or induces unpleasant side effects such as nausea and a dry mouth leading to an indirect reduced food intake in rats remains unclear at the moment. So far, it could already be shown, that imipramine leads to a reduction of salivary flow in rats but in a test to predict the emetic potential of drugs, imipramine did not seem to induce this [48, 49].

Many publications have previously shown that conducting the FST in rats leads to an elevation of plasma corticosterone, the primary stress hormone in rats [16, 50–52]. Only a few studies examined how long the stress response lasted after the FST [53, 54]. One group could show a recovery after 135 min in male animals [54]. Furthermore, in some studies, corticosterone levels were measured by repeated blood analyses or did not employ a baseline measurement, which might have also confounded the results [55, 56]. In our study, we conducted a non-invasive approach to examine the duration of elevated corticosterone levels by measuring fecal corticosterone metabolites (FCMs) over the whole time course of the FST and afterwards [57]. By choosing different sample intervals, we tried to represent the time of the acute stress response and the time of recovery.

Interestingly, we only observed significantly higher FCM concentrations in the FST-Imi group in both sexes after the forced swim test, but not in the other groups. This finding contrasts with Pintér and colleagues [16], who did not find an altered corticosterone response after imipramine administration. The same study also showed that water temperature does not seem to influence the amplitude of corticosterone release [16]. Additionally, early life experiences affect corticosterone levels after stress [58]. This aspect seems particularly important for the later cumulative evaluation of distress associated with the experiment. Finally, another study demonstrated that rats housed in an enriched environment had a lower corticosterone response after the FST than those in a non-enriched climate [59].

We detected much higher FCM levels in the second female cohort leading to strong cohort effects, which we cannot explain entirely. Circannual variations of the serum corticosterone concentrations might have partially influenced the results because the female cohorts were tested during different times of the year [60].

The forced swim test and forced swimming procedures are psychological stressors because the animals are experiencing an unescapable situation. We were surprised to see no effect in the saccharin preference test as a measure for anhedonia [61]. From this we conclude, that the stress induced by the standard FST protocol is probably too mild and too brief to cause anhedonia, a core symptom of major depressive disorder. This interpretation is reinforced by an earlier study subjecting rats to repeated forced swim stress in cold water, which induced a significantly lower preference for a sucrose solution [62].

The analysis of species-specific behaviors such as burrowing, nest score, and LINT did not reveal any effects of the FST on the wellbeing of the animals. The burrowing results in the female rats indicate that animals are not exhausted after the FST, as often criticized by animal rights organizations after the FST, and still burrow voluntarily shortly after the swimming procedure. However, we found that burrowing is not always a reliable parameter. Forty-four percent of the rats in the male cohort did not reach the threshold of the inclusion criteria (500 g in 60 min), so the experiment had to be discontinued. Several studies report that approximately 10–20% of rats do not show good burrowing behavior at baseline measurements [36, 63]. A recent study demonstrated profound strain differences in burrowing behavior [25]. Based on these findings, one should carefully consider the choice of the rat strain in future burrowing studies. Also, testing the rats during their active phase (i.e., at night), as usually done in mice, might increase burrowing behavior [23].

The fact that nest complexity was not affected by exposure to the FST is an additional indicator for the severity of the FST not being as high as discussed. But we cannot exclude the possibility of the nest score parameter not being sensitive enough to detect potential negative effects of the FST.

Regarding locomotion and exploratory behavior, we would have expected a reduction of the total distance moved in the open field resulting from the physical dimension of the FST. Instead, we found that the FST and/or treatment with imipramine does not alter open field parameters after two days. We assume that the latency until interaction with the nest material test (LINT) was not changed because it is also dependent on locomotion and exploration. One reason might be that the time between forced swim testing and the welfare parameters was too long to detect acute effects. Yet this this shows that any possible burden does not last longer than two days.

A limitation of our study is that we only tested one rat strain. One study could show different endocrine responses after the FST in rat strains [44, 64]. Therefore, a strain-dependent severity of the FST might be possible and should be examined in future studies.

Further factors influencing the experimental outcome might be the animals’ age, experimenters, handling procedures and the intensity of habituation to handling since this has already been demonstrated in the mouse [65–67]. Consequently, a study across labs could be very interesting due to the many influencing factors. Furthermore, an investigation of the severity of the FST in mice might be helpful to also give recommendations on the animal species on an animal welfare basis.

Additionally, the usage of potentially more sensitive parameters, e.g., telemetric recordings of heart rate and especially heart rate variability, focusing on the recovery of physiological variables of the animals might be an additional informative future approach.

The current treatment options for major depressive disorder are limited, and approximately one-third of the patients do not reach remission, highlighting the need for more research [68]. Until today, alternative testing methods, especially in the field of neurobiology, unfortunately, cannot replace animal experimentation to a full extent [69].

### Conclusion

So far, our experiments demonstrate that hypothermia is the only burden induced by the FST. Animals additionally treated with imipramine also showed mild body weight loss, reduced food intake, and elevated fecal corticosterone metabolite concentrations for three days after the FST. From our results, we conclude that the standard FST procedure itself only produces short-term moderate suffering and distress because of the profound but only short-lasting hypothermia and suggest a classification as ‘moderate’ according to the Directive 2010/63/EU. The ideal classification would be via a systematic approach comparing the relative severity of different models as previously performed in mice [70].

Applying pharmacological substances usually adds some discomfort to the animal and is always a part of severity classification in the process of the approval. In this case, the effect of the i.p. injection of imipramine was detected to have a stronger influence on the severity-associated measures than the swimming procedure. A promising refinement factor is using warmer water to allow the rats to maintain their physiological body temperature and prevent hypothermia. It might result in a procedure causing only mild stress in the animals.

## Supporting information

S1 File (DOCX)
