# Supplementary material for: Evidence-based severity assessment of the forced swim test in the rat
Source: PLoS One. 2023 Oct 12;18(10):e0292816. doi: 10.1371/journal.pone.0292816 (PMC10569541; doi:10.1371/journal.pone.0292816)
Supplement: S1 File — (DOCX) [file pone.0292816.s001.docx]

Supplementary Material

## Supplementary Figures


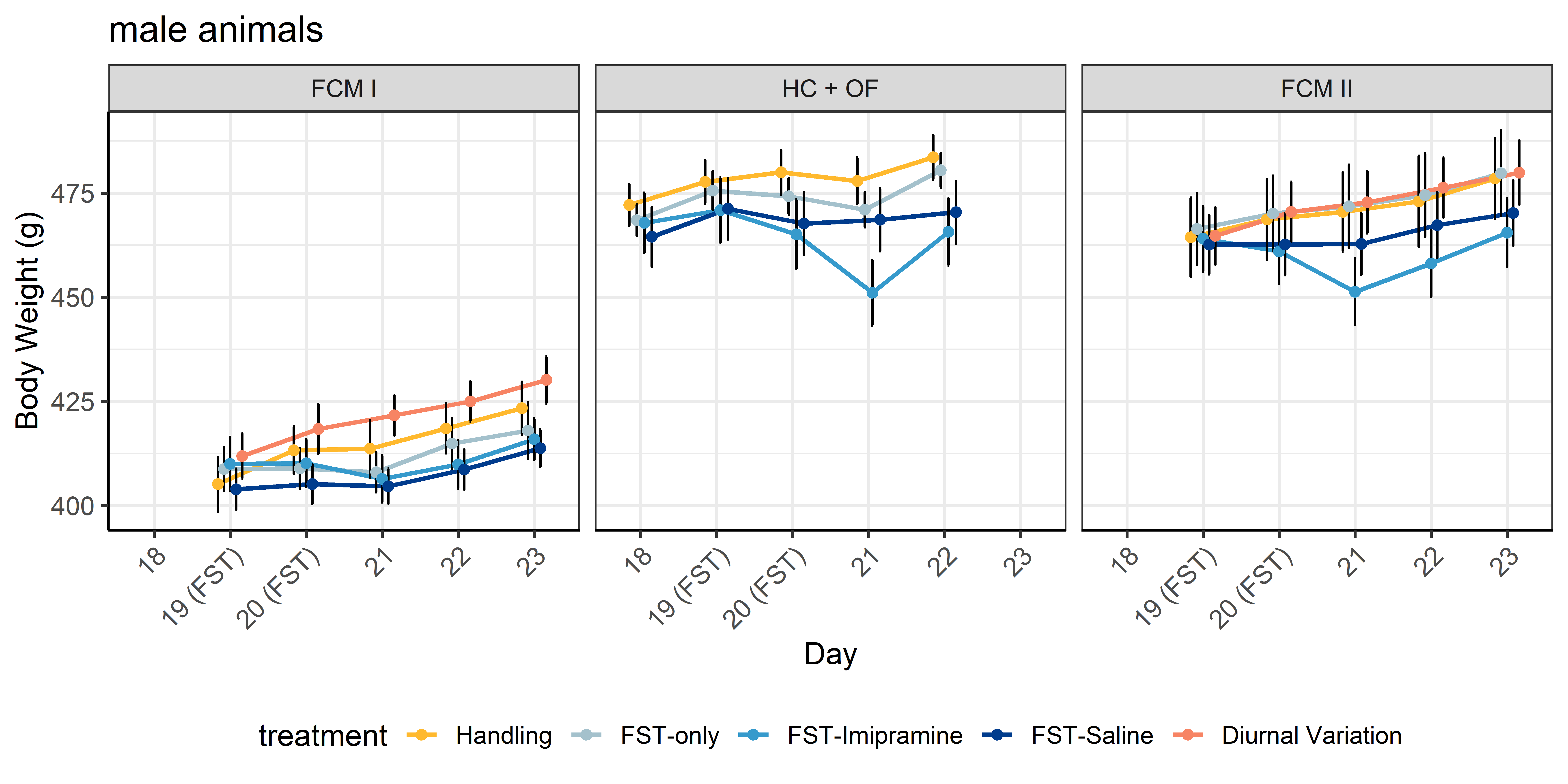


**S1 Figure:** Time course of the body weight in male animals of the respective cohorts. Data presented as mean ± SEM. Group sizes were n=8-12 animals.


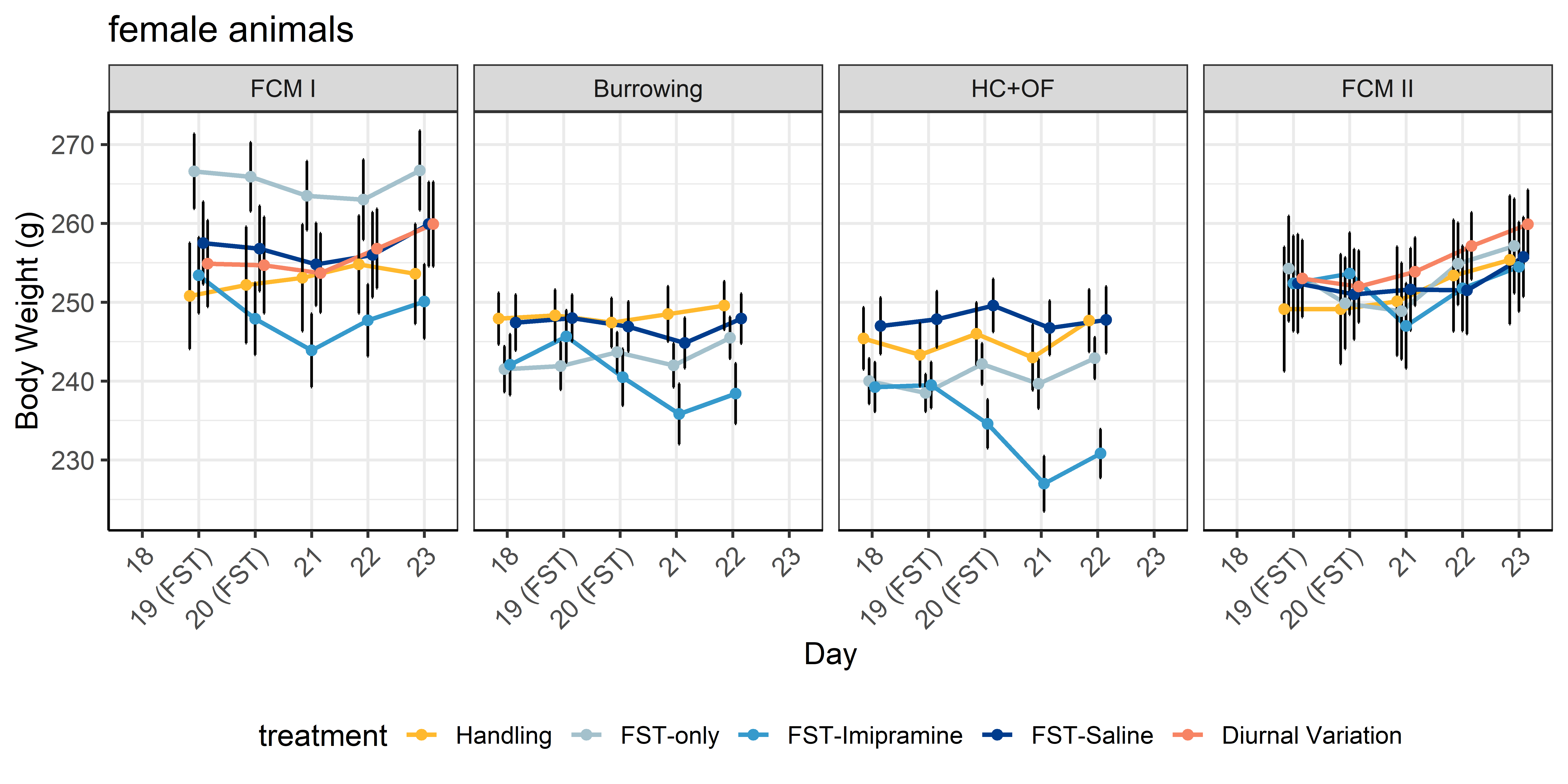


**S2 Figure:** Time course of the body weight in female animals of the respective cohorts. Data presented as mean ± SEM. Group sizes were n=8-12 animals.


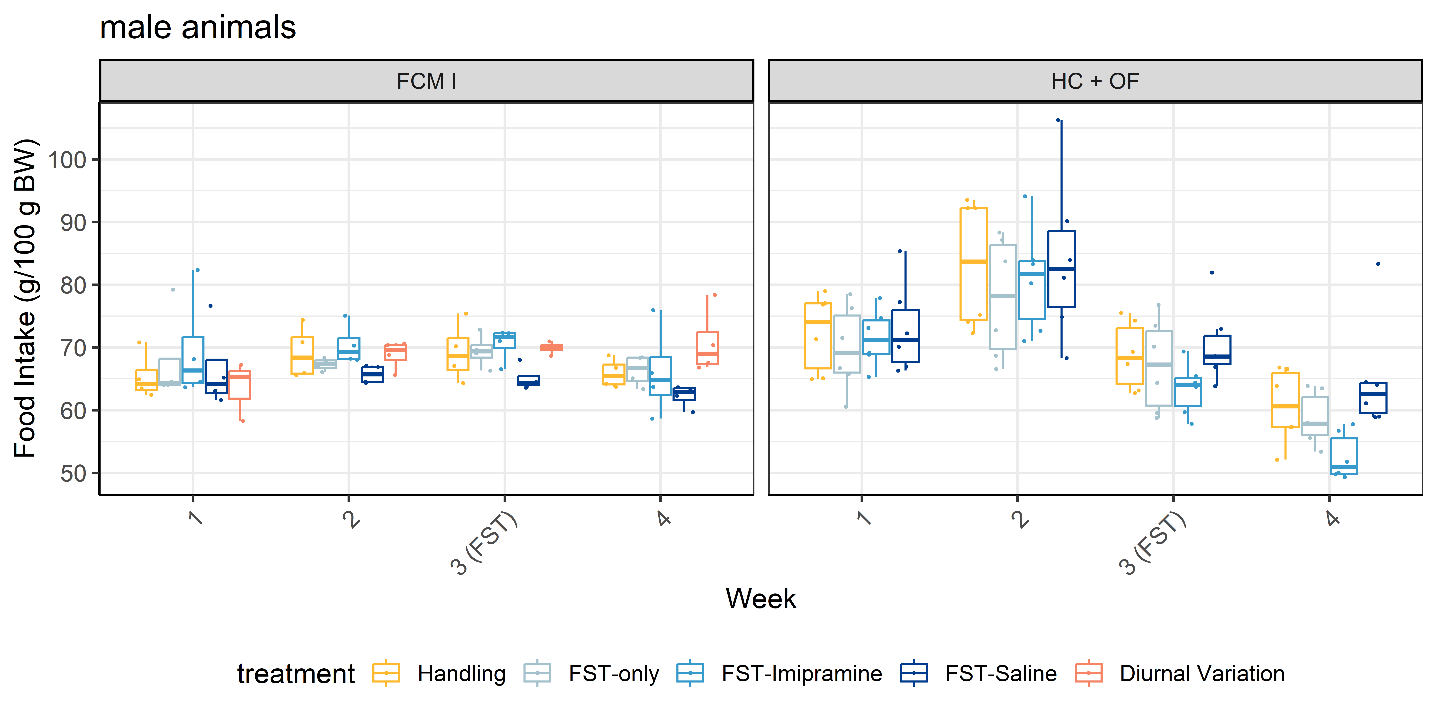


**S3 Figure:** Food consumption measured once a week in male animals of the first FCM cohort and the homecage cohort. Data is presented as boxplots. Group sizes were n=4-6 cages.


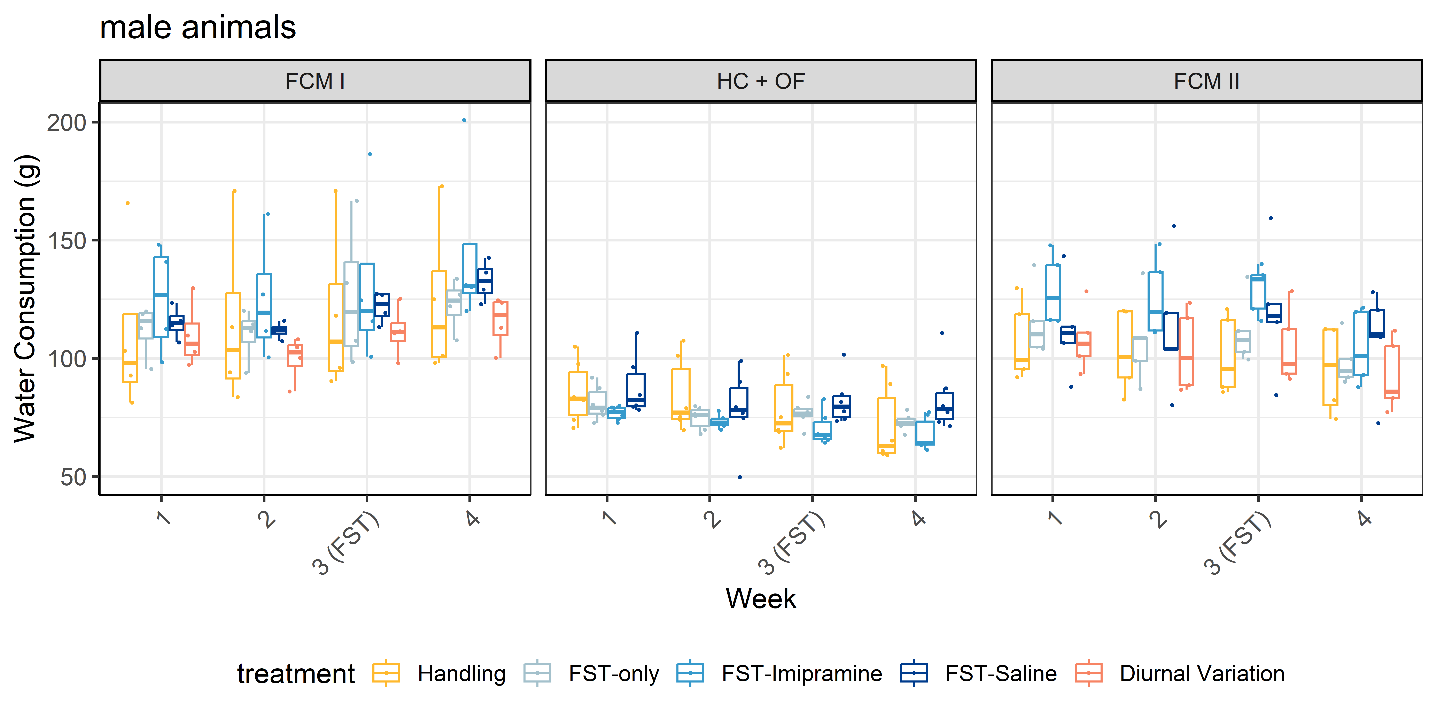


**S4 Figure:** Water intake measured once a week in male animals of both FCM cohorts and the homecage cohort. Data is presented as boxplots. Group sizes were n=4-6 cages.


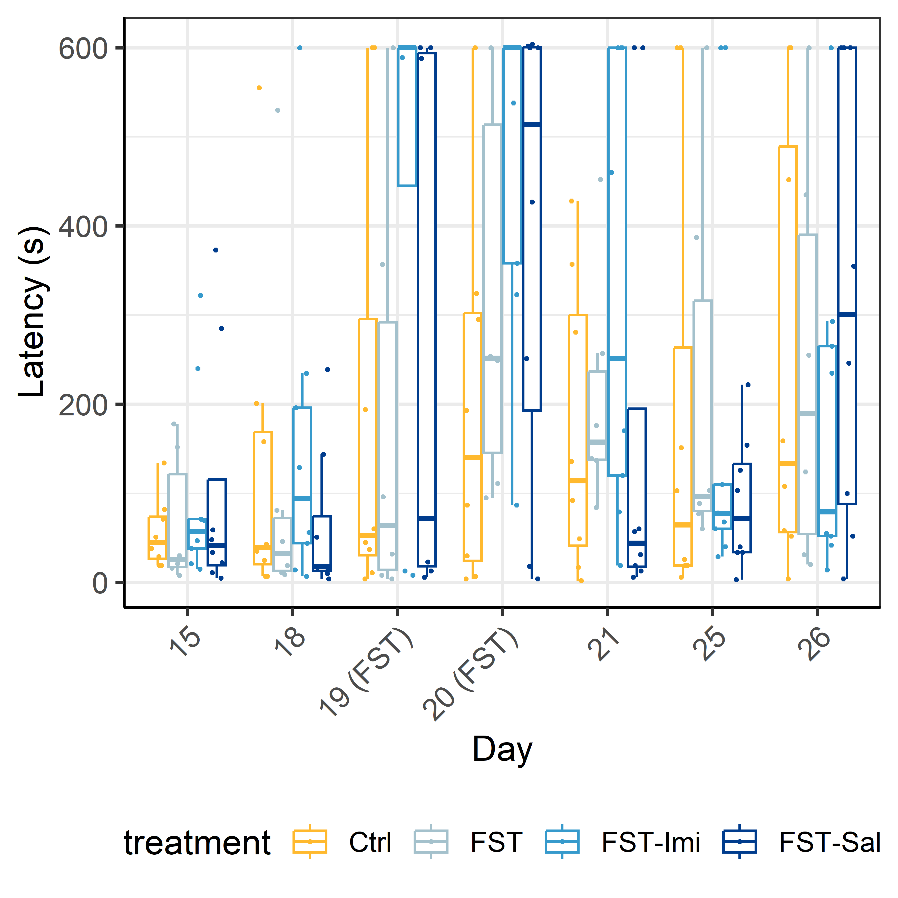


**S5 Figure:** Latency until start of burrowing in female rats. Data is presented as boxplots. Twelve animals were allocated to each treatment group.


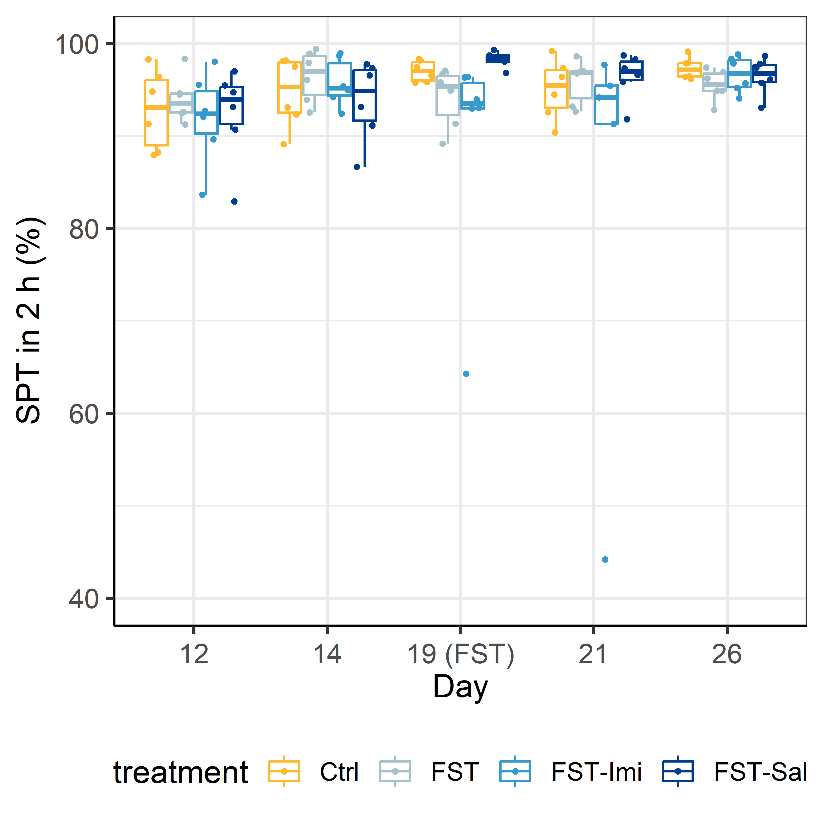


**S6 Figure:** Preference of saccharin over water measured during 2 h. Data is presented as boxplots. Group sizes were n=5-6 cages. SPT = Saccharin Preference Test.

## Supplementary results

**S1 Table:** Body Weight

| Sex | Cohort | Time effect | Treatment effect | Time*treatment interaction |
| --- | --- | --- | --- | --- |
| ♂ | FCM I | F(4, 175)=1.5, p=0.2 | F(4, 175)=0.37, p=0.83 | F(16, 175)=0.22, p=1 |
|  | HC + OF | F(4, 220)=0.43, p=0.79 | F(3, 220)=0.24, p=0.87 | F(12, 220)=0.46, p=0.93 |
|  | FCM II | F(4, 225)=0.39, p=0.82 | F(4, 225)=0.03, p=1 | F(16, 225)=0.15, p=1 |
| ♀ | FCM I | F(4, 225)=0.08, p=0.99 | F(4, 225)=1.25, p=0.29 | F(16, 225)=0.13, p=1 |
|  | Burrowing | F(4, 220)=0.06, p=0.99 | F(3, 220)=1.1, p=0.35 | F(12, 220)=0.42, p=0.96 |
|  | HC + OF | F(4, 220)=0.32, p=0.86 | F(3, 220)=1.28, p=0.28 | F(12, 220)=0.77, p=0.68 |
|  | FCM II | F(4, 175)=0.23, p=0.92 | F(4, 175)=0.1, p=0.98 | F(16, 175)=0.09, p=1 |

**S2 Table:** Weekly food intake

| Sex | Cohort | Time effect | Treatment effect | Time*treatment interaction |
| --- | --- | --- | --- | --- |
| ♂ | FCM I | F(3, 59)=0.89, p=0.45 | F(4, 59)=0.98, p=0.43 | F(12, 59)=0.78, p=0.67 |
|  | HC + OF | F(3, 80)=9.38, p<0.001 | F(3, 80)=0.2, p=0.9 | F(9, 80)=0.55, p=0.83 |

**S3 Table:** Weekly water consumption

| Sex | Cohort | Time effect | Treatment effect | Time*treatment interaction |
| --- | --- | --- | --- | --- |
| ♂ | FCM I | F(3, 60)= 0.23, p=0.88 | F(4, 60)=0.26, p=0.9 | F(12, 60)=0.1, p=1 |
|  | HC + OF | F(3, 80)=1.97, p=0.13 | F(3, 80)=1.22, p=0.3 | F(9, 80)=0.46, p=0.89 |
|  | FCM II | F(3, 79)=0.35, p=0.79 | F(4, 79)=1.28, p=0.28 | F(12, 79)=0.25, p=1 |
| ♀ | FCM I | F(3, 80)=0.95, p=0.42 | F(4, 80)=1.41, p=0.24 | F(12, 80)=0.12, p=1 |
|  | Burrowing | F(3, 79)=0.39, p=0.76 | F(3, 79)=0.03, p=0.99 | F(9, 79)=0.12, p=1 |
|  | HC + OF | F(3, 80)=1.84, p=0.15 | F(3, 80)=1.26, p=0.29 | F(9, 80)=0.38, p=0.94 |

**S4 Table:** Latency to start burrowing

| Sex | Cohort | Time | Kruskal-Wallis-Test |
| --- | --- | --- | --- |
| ♀ | Burrowing | D15 | χ²=1.02, p=0.8 |
|  |  | D18 | χ²=1.9, p=0.59 |
|  |  | D19 | χ²=3, p=0.39 |
|  |  | D20 | χ²=5.8, p=0.12 |
|  |  | D21 | χ²=4.5, p=0.21 |
|  |  | D25 | χ²=1.5, p=0.69 |
|  |  | D26 | χ²=1.01, p=0.79 |

**S5 Table:** Nest Score (Wilcoxon-Test)

| Sex | Cohort | Treatment Group | Time Points:  D13 vs. D20 | Time Points:  D14 vs. D21 |
| --- | --- | --- | --- | --- |
| ♂ | HC+OF | Ctrl | p=0.4142 | p=0.5716 |
|  |  | FST | p=0.4962 | p=0.3447 |
|  |  | FST-Imi | P=0.8241 | p=0.5862 |
|  |  | FST-Sal | p=0.5807 | p=0.7656 |
| ♀ | HC+OF | Ctrl | p=0.3304 | p=1 |
|  |  | FST | p=0.3447 | p=0.233 |
|  |  | FST-Imi | p=1 | p=1 |
|  |  | FST-Sal | p=0.2652 | p=0.08897 |

**S6 Table:** Lateny until interaction with nest material test (LINT)

| Sex | Cohort | Time Point | Kruskal-Wallis-Test |
| --- | --- | --- | --- |
| ♂ | HC + OF | D7 | Χ²=2.76, p=0.43 |
|  |  | D14 | Χ²=1.602, p=0.66 |
|  |  | D21 | Χ²=5.28, p=0.153 |
|  |  | D28 | Χ²=2.11, p=0.55 |
| ♀ | HC + OF | D7 | Χ²=1.51, p=0.68 |
|  |  | D14 | Χ²=1.69, p=0.64 |
|  |  | D21 | Χ²=7.78, p=0.051 |
|  |  | D28 | Χ²=0.63, p=0.89 |

**S7 Table:** Detailed Statistics of Body Weight Change

| Sex | Cohort | time*treatment interaction | Post-hoc (Tukey) |
| --- | --- | --- | --- |
| ♂ | FCM 1 | F(20, 210)=2.9, p<0.001 | Day 20:  Ctrl. vs. FST p=0.0003  Ctrl. vs. FST-Imi p=0.0003  Ctrl. vs. FST-Sal = p=0.0026  FST-only vs. DV p=0.0098  FST-Imi vs. DV p=0.01  Day 21:  FST-Imi vs. DV p=0.0026 |
|  | FCM 2 | F(24, 315)=3.8, p<0.001 | Day 20:  Ctrl. vs. FST-Imi p=0.0031  FST vs. FST-Imi p=0.0098  FST-Imi vs. DV p=0.0002  FST-Sal vs. DV p=0.02  Day 21:  Ctrl. vs. FST-Imi p<0.0001  FST vs. FST-Imi p<0.0001  FST-Imi vs. DV p<0.0001 |
|  | HC + OF | F(18, 308)=9.8, p<0.001 | Day 20:  Ctrl. vs. FST-Imi p<0.001  Ctrl. vs. FST-Sal p=0.0047  FST vs. FST-Imi p=0.04  FST vs. FST-Sal p<0.001  Day 21:  Ctrl. vs. FST-Imi p<0.001  FST vs. FST-Imi p<0.001  FST-Imi vs. FST-Sal p<0.001  Day 22:  Ctrl. vs. FST-Imi p<0.001  FST vs. FST-Imi p=0.006  FST-Imi vs. FST-Sal p<0.001 |
| ♀ | FCM 1 | F(16, 225)=2.1, p=0.008 | Day 20:  Ctrl. vs. FST-Imi p=0.0049  DV vs. FST-Imi p=0.066  Day 21:  Ctrl. vs. FST-Imi p=0.056 |
|  | FCM 2 | F(9, 176)=3.2, p=0.001 | Day 20:  FST vs. FST-Imi p=0.047  Day 21:  Ctrl. vs. FST-Imi p=0.0019  FST vs. FST-Imi p=0.047  FST-Imi vs. FST-Sal p=0.0035  FST-Imi vs. DV p=0.0004 |
|  | Burrowing | F(9, 176)=3.1, p=0.001 | Day 20:  Ctrl. vs. FST-Imi p=0.01  Day 21:  Ctrl. vs. FST-Imi p=0.0004  FST vs. FST-Imi p<0.001 |
|  | HC + OF | F(16, 175)=2.7, p<0.001 | Hand vs. Imi: 20 p=0.0002, 21 p=0.03  FST-only vs. Imi: 20 p<0.001, 21 p=0.02  Sal vs. Imi: 20 p=0.001, 21 p=0.02 |
